# Multicenter Study of Laparoscopic Common Bile Duct Exploration for Choledocholithiasis in the English-Speaking Caribbean

**DOI:** 10.7759/cureus.42949

**Published:** 2023-08-04

**Authors:** Shamir O Cawich, Sahle P Griffith, Charles W Greenidge, Kimon Bonadie, Fawwaz Mohammed, Greg M Padmore, Tameshwar Algu, Hemraj Ramcharran, Vijay Naraynsingh

**Affiliations:** 1 Surgery, University of the West Indies, St. Augustine, TTO; 2 Surgery, Queen Elizabeth Hospital, Bridgetown, BRB; 3 Surgery, St Jude Hospital, Vieux Fort, LCA; 4 Surgery, Tapion Hospital, Castries, LCA; 5 Surgery, Cayman Islands National Hospital, Grand Cayman, CYM; 6 Surgery, Port of Spain General Hospital, Port of Spain, TTO; 7 Surgery, New Amsterdam Public Hospital, Berbice, GUY; 8 Surgery, Georgetown Public Hospital Corporation, Georgetown, GUY; 9 Surgery, Medical Associates Hospital, St. Joseph, TTO

**Keywords:** extraction, cholangiogram, exploration, choledocholithiasis, laparoscopy

## Abstract

Background

Common bile duct (CBD) exploration to address choledocholithiasis is not widely practiced in the English-speaking Caribbean. This study sought to determine the frequency of laparoscopic CBD explorations in the English-speaking Caribbean and to document the stone clearance rates and short-term outcomes of this procedure.

Methods

We accessed records for all practicing laparoscopic surgeons in the English-speaking Caribbean who performed laparoscopic CBD explorations over a 10-year period from January 1, 2013, to June 30, 2023. The following data were extracted retrospectively from patient records: demographic details, operating time, stone clearance rates, retained stone rates, conversions, and complications. All data were analyzed with SPSS version 20 (IBM Corp., Armonk, NY).

Results

Over the 10-year study period, 35 patients underwent laparoscopic cholecystectomy and synchronous CBD exploration in Barbados, Cayman Islands, Guyana, Grenada, St Lucia, and Trinidad & Tobago. The procedure was performed at low volumes of only 0.7 procedures per surgical team per annum. The conversion rate to open CBD exploration was 13% and when laparoscopic CBD exploration was completed, it resulted in 96.3% stone clearance, 3.7% retained stones, mean hospitalization of two days, 9.7% minor morbidity, and no mortality.

Conclusion

Laparoscopic CBD exploration is feasible in the resource-poor Caribbean setting, and it yields good results, with 96.3% stone clearance rates, 9.7% minor morbidity, and no mortality. These results are better than those reported in Caribbean literature for stone extraction with endoscopic retrograde cholangiopancreaticography (ERCP).

## Introduction

Common bile duct (CBD) exploration to address choledocholithiasis was a routine procedure in the era of open surgery but is less popular among laparoscopic surgeons [1]. A similar scenario exists in Caribbean practice, where surgeons are generally reluctant to perform CBD exploration at the time of laparoscopic cholecystectomy, instead deferring to two-staged treatment with pre-operative endoscopic retrograde cholangiopancreaticography (ERCP) prior to laparoscopic cholecystectomy [2].

In a few centers in the English-speaking Caribbean, laparoscopic CBD explorations are performed for stone extraction. This study sought to determine the frequency of laparoscopic CBD explorations for choledocholithiasis in the English-speaking Caribbean and to document the stone clearance rates and short-term outcomes with this procedure.

## Materials and methods

The Anglophone Caribbean consists of 17 countries, with a cumulative population of 6,426,914 persons [3]: Anguilla, Antigua & Barbuda, Bahamas, Barbados, Belize, British Virgin Islands, Cayman Islands, Dominica, Grenada, Guyana, Jamaica, Montserrat, St. Kitts & Nevis, St. Lucia, St. Vincent & the Grenadines, Trinidad & Tobago, and Turks & Caicos. All surgeons in these nations are fellows of the Caribbean College of Surgeons (CCOS), founded in 2002 as a professional association to promote surgical education for general surgeons practicing in the Anglophone Caribbean [3]. Therefore, approval for this study was sought from and granted by the CCOS.

We contacted the CCOS’ general membership by email and/or telephone to identify practicing surgeons who performed laparoscopic CBD exploration for choledocholithiasis. Surgeons performing laparoscopic CBD explorations were invited to participate by reporting their data. All data were cross-checked by retrospectively examining records from operating theaters in each of the English-speaking Caribbean countries over a 10-year period from January 1, 2013, to June 30, 2023. Any patient who underwent laparoscopic CBD exploration was identified and their records were retrieved for detailed analysis. The following data were extracted: demographic details, operating time, stone clearance rates, retained stone rates, conversions, and complications. All data were entered into a Microsoft Excel database (Microsoft Corporation, Redmond, WA) and the data were analyzed with SPSS version 20 (IBM Corp., Armonk, NY).

We defined stone clearance as the removal of all stones within the CBD after duct manipulation, confirmed on cholangiography or choledochoscopy. A retained stone was considered as one that was detected in the CBD less than six months after cholecystectomy [4]. Recurrent CBD stones were defined as those detected more than six months following cholecystectomy [4].

## Results

Over the 10-year study period, 35 patients underwent laparoscopic cholecystectomy and synchronous CBD exploration. There were 31 females and 4 males at a mean age of 47.1 years (Range 23-68; SD ±11.9; Median 49). The teams performing laparoscopic CBD exploration were located in Barbados, Cayman Islands, Guyana, Grenada, St Lucia, and Trinidad & Tobago. Thirty-four patients underwent conventional laparoscopic explorations and 1 underwent FreeHand® robot-assisted laparoscopic exploration. Paper-based records could not be located for four patients, and these cases were excluded from further analysis. The final study sample comprised 31 patients who underwent laparoscopic CBD explorations.

**Figure 1:**
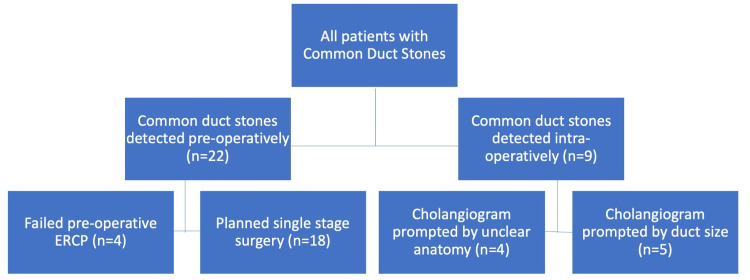
Patients Undergoing Laparoscopic Cholecystectomy, Operative Cholangiography, and Common Duct Exploration for Stone Extraction

In this setting, routine cholangiography was not practiced. We performed selective cholangiography when prompted by the presence of jaundice or deranged liver function tests. In 22 (71%) patients, choledocholithiasis was diagnosed preoperatively on imaging as prompted by clinical symptoms and/or abnormal liver function tests, and the diagnosis was only made intraoperatively in nine (29%) patients (Figure 1). The average CBD stone burden was 4.5 stones (Range 1-12; SD ±3.9; Median 3), but the stone size was not consistently recorded.

Exploration of the CBD was attempted via the trans-cystic route in 15 cases (the mean stone burden in this group was 4.6 stones per patient), but four of these patients required a choledochotomy when stones could not be retrieved by the trans-cystic route. In total, 20 patients had exploration via choledochotomy (the mean stone burden in this group was 3.43 stones per patient). In these patients, CBD stones were extracted using a combination of approaches, including Fogarty or Foley catheters (18), Dormia baskets (11), and stone advancement (6) using irrigation and/or a choledochoscope. The biliary tree was repaired with laparoscopic sutures in all cases, and T-tubes were only utilized in three (9.7%) cases.

There were four (13%) conversions to open CBD exploration for failed attempts at laparoscopic stone extraction. In this group, the patients had stones proximal to the cystic duct junction (2) and heavy stone burden (>8 stones) in the CBD (2). The surgeons completed laparoscopic CBD exploration in the remaining 27 (87.1%) cases.

A total of 27 patients had laparoscopic CBD exploration completed. Table 1 documents the clinical outcomes in this group of patients.

**Table 1:** Clinical Outcomes After Laparoscopic Cholecystectomy, Cholangiography, and Common Bile Duct Exploration (n=27)

Clinical Outcomes after Laparoscopic Cholecystectomy, Cholangiography & Common Bile Duct Exploration (n=27)	
Parameter	N (%)
Mean operating time	217.7 minutes; Range 90-480; SD ±90.1; Median 190
Mean hospitalization	2 days; Range 0-12; SD ±2.9; Median 1
Stone clearance	26 (96.3%)
Mortality	0
Major complications	0
Minor complications	3 (9.7%)
- Bile leak (biliary)	2
- Pneumonia (non-biliary)	1
Retained stones	1 (3.7%)

Two (7.4%) patients in this series had bile leaks post-operation. In both patients, the bile leaks settled with drainage through 15 Fr Blake drains that were placed in Morrison's pouch at the time of operation. None of these patients required postoperative ERCP and/or surgical intervention.

One (3.7%) patient in this series had a retained stone. This was a 30-year-old female who had undergone a prior failed attempt at ERCP. At laparoscopy, two stones were retrieved via a choledochotomy. A cholangiogram was done post-exploration. The operative note documented that it was of “poor quality,” and the surgeon interpreted that the CBD was clear post-exploration. This patient was identified post-operation when she re-presented with jaundice and abnormal liver function tests.

## Discussion

In Caribbean practice, ERCP is not readily available [5]. A prior report from the CCOS documented that ERCP was readily available in only four (24%) countries in the Anglophone Caribbean up to the year 2023 [5]. Considering that choledocholithiasis is present in 3-10% of patients undergoing laparoscopic cholecystectomy [6], it is probably a skill that laparoscopic surgeons in the Caribbean should acquire.

In the Caribbean, many patients are transferred to the four nations in which ERCP is available for preoperative endoscopic stone extraction prior to laparoscopic cholecystectomy. This, however, may not be in the best interest of the patients, as they are exposed to two-staged treatment, multiple exposures to anesthesia, increased cost, and treatment delays. On the other hand, when Pan et al. compared preoperative ERCP and subsequent laparoscopic cholecystectomy to single-stage laparoscopic cholecystectomy with CBD exploration in 1,757 patients with CBDS across 13 trials, they demonstrated the inferiority of the two-staged treatment [7]. Those patients who underwent synchronous laparoscopic CBD exploration and cholecystectomy had greater stone clearance (94% vs 90%), lower treatment costs, lower morbidity (7.6% vs 12%), less retained stones (1.2% vs 7.9%), lower cumulative operating time (112 vs 132 minutes), and reduced hospitalization (4.9 vs 6.6 days) [7].

In addition, there are data from the Caribbean showing that patients who experience delays before laparoscopic cholecystectomy experience significantly more hospital readmissions for complications of gallstone disease [2,8,9], longer postoperative hospitalization [2,8], and longer duration of cholecystectomy [8]. There is also published data from the Caribbean to show that ERCP resulted in 48% [10] to 79.3% [11] stone clearance, 10% [10] to 11% [12] morbidity, 10% incidence of post-ERCP pancreatitis [10], and 1% procedural-related mortality [10]. The data from this multicenter study show that laparoscopic CBD exploration performs better than ERCP in stone clearance rates (96.3% vs 79.3%), post-procedural pancreatitis (0 vs 10%), and mortality (0 vs 1%). Moreover, the complication rates for single-stage treatment (9.7%) were similar to that of ERCP alone (10-11%).

The 96.3% stone clearance rate in this multicenter study was comparable to existing reports in surgical literature [13-18] where stone clearance rates after laparoscopic CBD exploration were reported to range from 85% [13-14] to 97% [18]. It is important to note that these explorations were performed with minimal additional hardware than required for laparoscopic cholecystectomy. This is relevant because, in a prior survey of CCOS surgeons [5], it was reported that 91% believed that their “operating theatres were not equipped to perform laparoscopic CBD explorations” and 100% believed that “laparoscopic CBD explorations had high failure rates”. The results of this multicenter study in Caribbean practice directly contradict these opinions.

In addition to stone clearance, the morbidity and mortality in this multicenter study were comparable to existing reports in the surgical literature documenting <10% morbidity and <1% mortality [13,14,19]. The operating time in this report (217 minutes) was longer than many reports in the surgical literature, ranging from 120 minutes [16] to 194 minutes [20]. We must point out, however, that these procedures were performed infrequently in the Caribbean. These 35 explorations were performed over 10 years by five teams - a mean annual volume of only 0.7 cases per surgical team. We anticipate that the operating time should improve as the procedure is embraced by Caribbean surgeons and the case volumes increase.

We acknowledge that laparoscopic CBD exploration does require surgical expertise, the ability to interpret biliary anatomy on cholangiography, and some additional equipment, but our study demonstrates that it is feasible in the Caribbean environment. We believe it is important for Caribbean surgeons to be aware that the procedure is feasible. Current and future surgeons in the Caribbean environment may also benefit from mentoring with surgeons who perform these procedures in the region.

## Conclusions

Laparoscopic CBD exploration is feasible in the resource-poor Caribbean setting and it yields good results, with 96.3% stone clearance rates, 9.7% minor morbidity, and no mortality. These results are better than those reported in Caribbean literature for stone extraction with ERCP.
